# Association between circulating 25-hydroxyvitamin D concentrations and hip replacement for osteoarthritis: a prospective cohort study

**DOI:** 10.1186/s12891-021-04779-4

**Published:** 2021-10-19

**Authors:** Sultana Monira Hussain, Yuanyuan Wang, Alicia K. Heath, Graham G. Giles, Dallas R. English, Darryl W. Eyles, Elizabeth J. Williamson, Stephen E. Graves, Anita E. Wluka, Flavia M. Cicuttini

**Affiliations:** 1 Department of Epidemiology and Preventive Medicine, School of Public Health and Preventive Medicine, Monash University, 553 St Kilda Road, Melbourne, VIC 3004 Australia; 2 Department of Epidemiology and Biostatistics, School of Public Health, Imperial College London, London, W2 1PG UK; 3 Centre for Epidemiology and Biostatistics, Melbourne School of Population and Global Health, The University of Melbourne, Carlton, VIC 3053 Australia; 4 Cancer Epidemiology Division, Cancer Council Victoria, Melbourne, VIC 3004 Australia; 5 Queensland Brain Institute, The University of Queensland, Brisbane, QLD 4072 Australia; 6 Queensland Centre for Mental Health Research, The Park Centre for Mental Health, Wacol, QLD 4076 Australia; 7 Department of Medical Statistics, London School of Hygiene & Tropical Medicine, London, WC1E 7HT UK; 8 Department of Surgery, Flinders University, Bedford Park, SA 5042 Australia; 9 Australian Orthopaedic Association National Joint Replacement Registry, Discipline of Public Health, School of Population Health & Clinical Practice, University of Adelaide, Adelaide, SA 5005 Australia

## Abstract

**Background:**

To examine the association between circulating 25(OH)D concentrations and incidence of total hip replacement for osteoarthritis in a prospective cohort study.

**Methods:**

This study examined a random sample of 2651 participants in the Melbourne Collaborative Cohort Study who had 25(OH)D concentrations measured from dried blood spots collected in 1990-1994. Participants who underwent total hip replacement for osteoarthritis between January 2001 and December 2018 were identified by linking the cohort records to the Australian Orthopaedic Association National Joint Replacement Registry. Cox proportional hazard regression was used to estimate hazard ratios (HR) and 95% confidence intervals (CI) of total hip replacement for osteoarthritis in relation to 25(OH)D concentrations, adjusted for confounders.

**Results:**

Eighty-six men and eighty-seven women had a total hip replacement for osteoarthritis. Compared with men in the lowest (1st) quartile of 25(OH)D concentration, the HR for total hip replacement was 2.32 (95% CI 1.05, 5.13) for those in the 2nd quartile, 2.77 (95% CI 1.28, 6.00) for those in the 3rd quartile, and 1.73 (95% CI 0.75, 4.02) for those in the highest quartile of 25(OH)D concentrations (*p* for trend 0.02). There was little evidence of an association in women.

**Conclusions:**

Higher circulating 25(OH)D concentrations were associated with an increased risk of total hip replacement for osteoarthritis in men but not in women. Although the underlying mechanism warrants further investigation, our findings highlight the need to determine the optimal levels of circulating 25(OH)D to reduce the risk of hip osteoarthritis.

## Background

Hip osteoarthritis (OA) causes pain and disability and is a common musculoskeletal condition that affects millions of people worldwide. The lifetime risk of symptomatic hip OA is 18.5% for men and 28.6% for women [1]. There are no effective treatments for hip OA that alter disease process; as a result the prevalence and burden of disease due to hip OA are increasing. Costly total hip replacements are used to treat symptomatic end-stage hip OA, but 7 to 23% of patients experience ongoing pain despite technically successful surgery [2]. Therefore the strategy for reducing the burden of hip OA lies in shifting the management paradigm from palliation to disease prevention and reduction of disease progression.

As the hip is a ball and socket joint, it is susceptible to altered hip bone shape and joint congruency, which are important risk factors for the development and progression of hip OA [3]. Vitamin D is critical in bone mineralization, remodelling, and maintenance and thus is capable of changing bone shape [4]. Serum or plasma 25-hydroxyvitamin D [25(OH)D] concentration is considered the best indicator of vitamin D status [5]. Previous studies have examined whether circulating 25(OH)D concentrations are associated with hip OA in populations at risk of osteoporosis [6, 7] or community-based populations [8–10]. Two studies reported that low serum levels of 25(OH)D were associated with increased prevalence and incidence of radiographic hip OA among men and women aged ≥65 years (mean age 73.5 years, standard deviation 5.8 years) [6, 7]. However, there is uncertainty regarding the generalizability of these results. In the Study of Osteoporotic Fractures, women who had low incidence of fracture were excluded [6, 11], and in the Osteoporotic Fractures in Men Study, 17% of participants reported a fracture in the year prior to recruitment [7, 12], indicating the presence of selection bias towards high risk of fracture in both studies. The three community-based cohort studies not primarily aimed at examining osteoporosis [8–10] are summarized in Table 1. One Finnish study of 805 participants found no association between circulating 25(OH)D concentrations and the incidence of hip OA, based on 40 cases of hip OA [10]. In contrast, another Finnish study of 5274 participants showed a trend of increased risk of hip OA associated with increasing circulating 25(OH)D concentrations, but this study was based on only 45 cases of hip OA [9]. In an Australian study of 9135 participants, we found that higher concentrations of circulating 25(OH)D were associated with increased risk of hip replacement for severe OA in men but not in women [8]. We used a robust definition of hip OA, i.e. hip replacement for OA, which in the context of the Australian healthcare system is a valid measure of severe hip OA, as all Australian citizens and permanent residents have access to quality health care services under Australia’s publicly funded universal health insurance system (Medicare). Furthermore, our study had a large number of joint replacements, increasing the power to show potential associations [8].

**Table 1:** Association between 25-hydroxyvitamin D and hip osteoarthritis: data from three studies of community-based populations

Author, Year, Country	Follow-up	Participants	Outcome measurement	Number of cases	Adjustment	Main findings 25(OH)D nmol/l; OR/RR/HR; 95% CI
Konstari, 2012, Finland [10]	22-23 years	805 Finnish adults from the Mini-Finland Health Examination Survey Age: ≥30 years 55.3% women	Hip OA was defined by -documented history of previously diagnosed hip OA -hip arthroplasty due to OA	17 men and 23 women	age, gender, education, BMI, physical workload, smoking, leisure time physical activity, injuries, and season of blood draw	Q1 (13-33): reference Q2 (34-43): 1.22; 0.46-3.21 Q3 (44-56): 1.15; 0.43-3.04 Q4 (57-180): 0.72; 0.24-2.13 *P* for trend: 0.23
Konstari, 2014, Finland [9]	10 years	5274 adults living in mainland Finland from the Health 2000 Survey Age ≥ 30 years 54.1% women	Incidence of hip OA was drawn from the National Health Care Register using International Classification of Diseases, 10th revision (ICD-10)	45 participants	Age, gender, BMI, current physical workload, education, smoking, leisure time physical activity, time of serum collection, injuries and difficulty in walking due to discomfort or trouble in the knee or hip	Q1 (0-33): reference Q2 (34-42): 2.23; 0.76-6.55 Q3 (43-54): 2.96; 1.07-8.21 Q4 (55-134): 2.81; 1.01-7.85 *P* for trend 0.053
Hussain, 2015, Australia [8]	9.1 ± 2.7 years	9135 participants from the Australian Diabetes, Obesity and Lifestyle Study including population representative of Australia Age: ≥40 years 54.3% women	Incidence of hip replacement for OA by linking cohort records to the Australian Orthopaedic Association National Joint Replacement Registry	90 men and 111 women	Age, BMI, ethnicity, smoking status, physical activity, season of blood collection, latitude, hypertension, diabetes, and Area-level disadvantage.	Men Q1 (≤51): reference Q2 (52-65): 2.02; 1.01-4.06 Q3 (66-81): 2.17; 1.08-4.06 Q4 (≥82): 2.30; 1.09-4.82 *P* for trend 0.04 Women Q1 (≤41): reference Q2 (42-54): 1.21; 0.70-2.09 Q3 (55-69): 1.08; 0.61-1.91 Q4 (≥70): 1.29; 0.71-2.34 *P* for trend 0.49

*OA* osteoarthritis, *25(OH)D* 25-hydroxyvitamin D, *Q* quartile, *OR* odds ratio, *RR* relative risk, *HR* hazard ratio, *CI* confidence interval

Given the major focus on targeting vitamin D for the prevention of osteoporosis, understanding the effect of vitamin D on the risk of hip OA will be important to inform public health recommendations. As there is uncertainty regarding the association between circulating 25(OH)D concentrations and hip OA, we used a different well-established cohort study, with participants recruited from the community independent of any musculoskeletal disease, and long-term follow-up with enriched data prospectively collected [13], to investigate whether circulating 25(OH)D concentrations are associated with the incidence of total hip replacement for severe OA.

## Methods

### Participants

The Melbourne Collaborative Cohort Study (MCCS) is a prospective cohort study of 41,513 participants aged 27-75 years, recruited via the electoral roll, advertisements and community announcements in local media between 1990 and 1994, aimed at investigating the roles of diet and lifestyle in cancer and other non-communicable diseases [13, 14]. The study protocol was approved by the Cancer Council Victoria Human Research Ethics Committee (IEC No. 9001). Participants gave written informed consent to participate and for the investigators to obtain access to their medical records.

A case-cohort study nested within the MCCS was set up to investigate 25(OH)D in relation to breast, prostate, and colorectal cancers, diabetes, and mortality. Eligibility for the case-cohort study was restricted to participants who had dried blood spot samples available and no diagnosis of cancer prior to recruitment (n eligible = 29,205). As part of the case-cohort study, a sex-stratified random sample of 2996 eligible participants was selected as the “subcohort”. The current investigation involves this random sample of subcohort participants. All methods were carried out in accordance with relevant guidelines and regulations.

### Identification of hip replacement

Participants with a primary total hip replacement were identified from the Australian Orthopaedic Association National Joint Replacement Registry (AOA NJRR). The Registry began data collection in September 1999 and implementation was introduced in a staged fashion in each of the Australian states and territories. Victoria commenced data collection in January 2001 and the AOA NJRR has collected national data on joint replacement procedures performed in Australia since 2002 [15]. The Registry has detailed information on the joint replacement prostheses, patient demographics, the reason for joint replacement, whether it is a primary joint replacement or a revision and the type of revision. Data are collected from both public and private hospitals and this is validated using a sequential multi-level matching process against State and Territory Health Department unit record data [15]. Following the validation process and retrieval of unreported records, the Registry collects an almost complete set of data relating to hip and knee replacements in Australia [15]. Identifying information for MCCS participants, including first name, last name, date of birth, and sex, was provided to the staff at the AOA NJRR in order to identify MCCS participants who had undergone a joint replacement between 1 January 2001 and 31 December 2018. The matching was performed on these data provided using the Freely Extensible Biomedical Record Linkage (Febrl) system. Exact matches were identified and probabilistic matches were reviewed. The data linkage study protocol was approved by the Human Research Ethics Committees of Cancer Council Victoria (HREC 0601), and Monash University (2006000608).

### Demographic, lifestyle factors, medical history, and anthropometrics

At recruitment, data on demographic (sex, date of birth, country of birth) and lifestyle factors (physical activity, smoking status), Socio-Economic Indexes For Areas (SEIFA) index of relative disadvantage in quintile groups, and medical history (diabetes and hypertension) were collected using questionnaires in face-to-face interviews [13]. Physical activity was assessed by three separate questions obtained from the Risk Factor Prevalence Study conducted by the National Heart Foundation and Australian Institute of Health regarding frequency of non-occupational vigorous and moderate physical activity, and walking [16, 17]. Participants were categorised as whether or not participating in vigorous activity in line with values published in the Compendium of Physical Activities [18]. Height and weight were measured according to written protocols based on standard procedures, and body mass index (BMI) was calculated [13]. Data on self-reported knee and hip replacement were collected at the second wave of active follow-up (2003-2007).

### Circulating 25(OH)D measurement

Blood samples were collected, and for participants recruited from the second year of recruitment onwards (i.e. for approximately 75% of participants), whole blood was spotted onto Whatman 903 paper. Spots were air dried and then stored in the dark. Circulating 25(OH)D concentrations were measured from dried blood spots using liquid chromatography-tandem mass spectrometry (LC-MS/MS) as previously described [19, 20]. The laboratory uses National Institute of Standards and Technology (NIST) Vitamin D Standard Reference Materials and participates in the Vitamin D External Quality Assessment Scheme (DEQAS). It has been shown that measurement of 25(OH)D in dried blood spots is a valid and reliable alternative to conventional serum or plasma measurement [19]. Importantly, a simple calibration model has been developed to convert dried blood spot measurements to equivalent plasma concentrations, facilitating comparisons against clinical reference ranges and with studies using sera or plasma samples [19]. Measured 25(OH)D concentrations in dried blood spots were corrected for batch effects and seasonal variation and converted to plasma-equivalent concentrations as previously described [21]. All results presented are for batch- and season-adjusted plasma-equivalent 25(OH)D concentrations.

### Statistical analysis

**Fig. 1:**
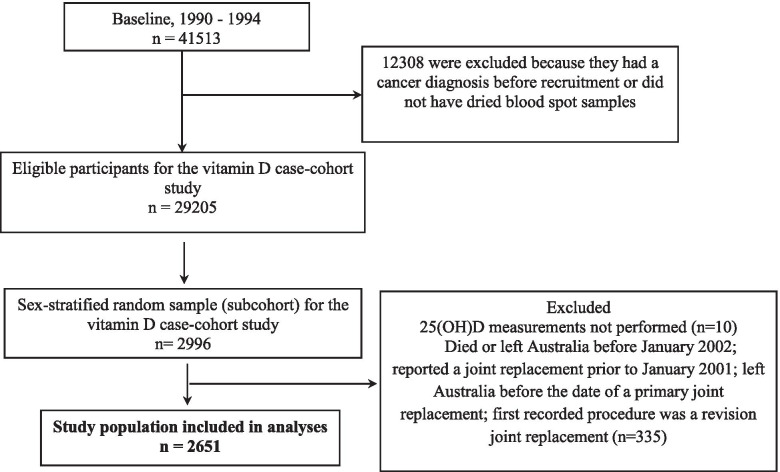
Flowchart of Melbourne Collaborative Cohort Study participants included in the analyses

Participants were excluded if 25(OH)D measurements were not performed (*n* = 10); they died or left Australia before January 2002; they reported at the second wave of follow-up (2003-2007) having had a joint replacement prior to 1 January 2001; left Australia before the date of a primary joint replacement; their first recorded procedure in the AOA NJRR was a revision joint replacement (*n* = 335) (Fig. 1).

Characteristics and the number of hip replacements in participants who were included in the current study and all other MCCS participants were compared. Characteristics of study participants were tabulated based on sex and hip replacement for OA. Cox proportional hazards regression analyses were used to estimate hazard ratios (HRs) and 95% confidence intervals (CIs) for hip replacement due to OA in relation to 25(OH)D concentrations, with age as the time scale. Follow-up for total hip replacement for OA (i.e. calculation of person-time) began on 1 January 2001, and ended at the date of first total hip replacement for OA or date of censoring. Participants were censored at the date of first total hip replacement performed for indications other than OA including hip fracture, avascular necrosis; the date of first total knee replacement; the date of death; the date the participant left Australia; or end of follow-up (i.e. 31 December 2018, the date that ascertainment of joint replacement by the AOA NJRR was complete); whichever came first. The linear association between circulating 25(OH)D concentrations and total hip replacement for OA was examined by fitting 25(OH)D as a continuous variable, and reported per 25 nmol/L increment. Circulating 25(OH)D concentrations were also categorized into sex-specific quartiles. The lowest quartile was used as the referent category. For tests of linear trend, participants were assigned the median value in each quartile, and the corresponding variable was modelled continuously.

To test whether the association between 25(OH)D concentrations and total hip replacement for OA was modified by sex, interactions were fitted and tested using the likelihood ratio test. Since there was evidence for an interaction (*p* = 0.06), all analyses were performed for men and women separately, and adjusted for BMI (kg/m^2^, modelled continuously), vigorous physical activity (any vs no), smoking status (former/current vs never), country of birth (Australia/United Kingdom vs Italy/Greece), socioeconomic disadvantage (quintiles of Socio-Economic Indexes for Areas [SEIFA], most deprived [quintiles 1-3] vs least deprived [quintiles 4-5]), and co-morbidities (yes vs no): diabetes and hypertension. All statistical analyses were performed using Stata 15.0 SE (StataCorp LP., College Station, TX, USA).

## Results

Participants of the current study were younger (53.7 ± 8.7 vs. 55.1 ± 8.6 years), more likely to be men (54.6% vs 39.1%), to be born in Australia and UK (84.7% vs 75.3%), to participate in vigorous physical activity (25.9% vs 21.1%), to be former/current smokers (54.0% vs 41.5%), to be in the upper two (least disadvantaged) quintiles of SEIFA (53.6% vs 45.6%), and less likely to have hypertension (17.7% vs 21.5%) and diabetes (2.5% vs. 3.5%), compared with MCCS participants who were not included in the analysis. There was no significant difference in prevalence of total hip replacement for OA between the two groups (6.5% vs. 5.7%).

One hundred and seventy three participants (86 men and 87 women) had a total hip replacement for OA from January 2001 until December 2018, a mean follow-up of 15.7 (SD 4.6) years. The characteristics of the participants are presented in Table 2. Compared with men without a total hip replacement, men with a total hip replacement were less likely to be in the lowest 25(OH)D quartile and more likely to be in the 3rd 25(OH)D quartile, more likely to be least deprived and participate in vigorous physical activity, and less likely to be current/former smokers. Women having a hip replacement did not noticeably differ with respect to levels of 25(OH)D or other variables with those who did not have a hip replacement.

**Table 2:** Characteristics of study participants at recruitment

	Men		Women	
	Total hip replacement for OA		Total hip replacement for OA	
	No	Yes	No	Yes
Number of participants	1379	86	1099	87
25(OH)D (nmol/L), median (IQR)	54.5 (42.7-69.0)	57.0 (47.3-66.4)	42.8 (34.7-52.7)	44.6 (34.5-53.3)
25(OH)D Quartile, n (%)				
1 (M < 43.00; F < 34.80 nmol/L)	354 (25.7)	12 (13.9)	279 (25.3)	23 (26.4)
2 (M 43.00- < 54.85; F 34.80- < 42.92 nmol/L)	346 (25.1)	24 (27.9)	278 (25.3)	19 (21.8)
3 (M 54.85- < 69.22; F 42.92- < 53.04 nmol/L)	337 (24.4)	31 (36.1)	272 (24.8)	23 (26.4)
4 (M ≥ 69.22; F ≥ 53.04 nmol/L)	342 (24.8)	19 (22.1)	270 (24.6)	22 (25.3)
Age (years), mean (SD)	53.5 (9.0)	55.4 (8.2)	53.3 (8.6)	56.6 (8.1)
Body mass index (kg/m^2^), mean (SD)	26.8 (3.5)	28.1 (3.2)	26.1 (5.0)	26.8 (4.4)
Country of birth, n (%)				
Australia/United Kingdom	1129 (81.9)	73 (84.9)	948 (86.3)	79 (90.8)
Italy/Greece	250 (18.1)	13 (15.1)	151 (13.7)	8 (9.2)
Socioeconomic status (SEIFA), n (%)				
Most deprived (quintiles 1-3)	678 (49.4)	37 (43.0)	475 (43.3)	38 (43.7)
Least deprived (quintiles 4-5)	694 (50.6)	49 (57.0)	621 (56.7)	49 (56.3)
Vigorous physical activity, n (%)	360 (26.1)	31 (36.1)	269 (24.5)	22 (25.3)
Smoking (former/current), n (%)	766 (55.6)	41 (47.7)	355 (32.3)	34 (39.1)
Diabetes, n (%)	58 (4.9)	2 (2.8)	22 (2.3)	1 (1.3)
Hypertension, n (%)	257 (21.5)	16 (22.5)	212 (21.8)	20 (25.6)

*OA* osteoarthritis, *25(OH)D* 25-hydroxyvitamin D, *IQR* interquartile range, *M* male, *F* female, *SD* standard deviation, *SEIFA* Socio-Economic Indexes for Areas

In men, the HR for total hip replacement for OA was 1.23 (95% CI 0.95-1.60) per 25 nmol/L increment in 25(OH)D concentration after adjusting for BMI, vigorous physical activity, smoking status, country of birth, and SEIFA (Table 3). Compared with men in the lowest (1st) quartile of 25(OH)D concentration, the HR for total hip replacement for OA was 2.06 (95% CI 1.02, 4.13) for those in the 2nd quartile, 2.40 (95% CI 1.21, 4.75) for those in the 3rd quartile, and 1.57 (95% CI 0.75, 3.29) for those in the highest quartile of 25(OH)D concentrations (*p* for trend 0.04). Additional adjustment for co-morbidities (diabetes and hypertension) did not change the results. In women, there was little evidence of an association (Table 3).

**Table 3:** Hazard ratios of total hip replacement for osteoarthritis in relation to circulating 25-hydroxyvitamin D concentrations

	Model 1^a^, HR (95% CI)	Model 2^b^, HR (95% CI)
Men		
25(OH)D (per 25 nmol/L increment)	1.23 (0.95-1.60) *p* = 0.12	1.26 (0.95-1.67) *p* = 0.11
25(OH)D Quartiles		
1 (<43.00 nmol/L)	Reference	Reference
2 (43.00 to <54.85 nmol/L)	2.06 (1.02-4.13)	2.32 (1.05-5.13)
3 (54.85 to <69.22 nmol/L)	2.40 (1.21-4.75)	2.77 (1.28-6.00)
4 (≥69.22 nmol/L)	1.57 (0.75-3.29)	1.73 (0.75-4.02)
*P for trend*	0.04	0.02
Women		
25(OH)D (per 25 nmol/L increment)	0.97 (0.64-1.47) *p* = 0.90	0.94 (0.60-1.47) *p* = 0.78
25(OH)D Quartiles		
1 (<34.8 nmol/L)	Reference	Reference
2 (34.8 to <42.92 nmol/L)	0.74 (0.40-1.37)	0.60 (0.32-1.16)
3 (42.92 to <53.04 nmol/L)	0.92 (0.51-1.66)	0.79 (0.43-1.47)
4 (≥53.04 nmol/L)	0.86 (0.47-1.57)	0.77 (0.41-1.44)
*P for trend*	0.93	0.34

*25(OH)D* 25-hydroxyvitamin D, *HR* hazard ratio, *CI* confidence interval

^a^adjusted for BMI, vigorous physical activity, smoking status, country of birth, and SEIFA

^b^adjusted for BMI, vigorous physical activity, smoking status, country of birth, SEIFA, and co-morbidities (diabetes and hypertension)

## Discussion

In this population-based cohort of middle-aged and older individuals, higher circulating 25(OH)D concentrations were associated with an increased risk of having a total hip replacement for OA in men but not in women, confirming our previous findings from an independent community-based cohort study [8].

In the Australian Diabetes, Obesity and Lifestyle Study of 9135 participants followed up for 9.1 (SD 2.7) years, we found an increased risk of having a hip replacement for OA with higher serum 25(OH)D concentrations in men but not women [8]. The current study examining an independent cohort, the MCCS, with a follow-up of 15.7 (SD 4.6) years, observed similar findings when hip OA was defined as hip replacement acquired from linking the cohort to NJRR. These linkage data are validated [22] and nearly complete (>99%) regarding joint replacements in Australia [15]. Our findings from the two Australian cohort studies are supported by findings from the Finland Health 2000 study which showed that higher concentrations of 25(OH)D were associated with an increased risk of developing hip OA [9]. However the Finnish study had only 45 cases of incident hip OA so was underpowered to examine sex differences [9].

The mechanism for the association between higher 25(OH)D concentrations and increased risk of hip replacement for OA is not clear. Vitamin D plays an important role in bone mineralization, remodelling and maintenance, and greater serum 25(OH)D concentrations are associated with increased bone mineral density [23, 24]. The effect of vitamin D on anabolic bone changes might lead to subchondral bone sclerosis and altered hip bone shape. There is increasing evidence that hip bone shape is modifiable and subtle changes in hip bone shape increase the risk of hip OA [3, 25], and that increased bone mineral density is a risk factor for hip OA [26]. The sex difference that we observed for the association between circulating 25(OH)D concentrations and severe hip OA may be mediated through circulating levels of testosterone and estrogen, since these hormones play a role in regulating vitamin D and calcium homeostasis [27]. Similarly, vitamin D is known to affect androgen synthesis in testicular cells in men [28], and possibly ovarian androgens and testosterone levels in women [29]. Other studies have also reported sex-dependent skeletal effects of vitamin D [30]. We have shown a link between sex steroid hormones and total hip replacement for OA [22, 31]. Sex differences in adiposity and body fat distribution might be another possible explanation. In our study, the HR for the association between 25(OH)D and THR was lower in the highest quartile compared with the HRs of the middle two quartiles, which is comparable to the biphasic relationship seen between 25(OH)D and chronic diseases such as cardiovascular disease [32], schizophrenia [33], and mortality [34]. However, any potential biphasic relationship between 25(OH)D and THR, if exists, needs to be interpreted with caution given the small numbers of observations of THRs in each of the 25(OH)D quartiles in our study. It might mean that genetic and environmental factors relating to 25(OH)D play a role in OA pathogenesis as has been seen in case of bone health [35].

This study has limitations. Although circulating 25(OH)D concentrations were measured for a random sample of MCCS participants, there were some differences between participants included in this study and the rest of MCCS participants. However, it is unlikely that the selection of participants for the current study would affect the relationship between 25(OH)D and risk of total hip replacement for OA. Whilst participants with 25(OH)D measured had better health and higher socioeconomic position compared with those without measurements, the risks of hip replacement were similar in the two groups. Circulating 25(OH)D concentrations were measured at a single time point (i.e. MCCS recruitment). There is good intra-individual consistency in 25(OH)D concentrations measured several years apart, thus a single measurement is a reasonable measure of long-term vitamin D status [36–38]. Furthermore, in middle-aged Caucasian adults the main determinants of circulating 25(OH)D concentration are sex, age, obesity, latitude, season of blood collection, physical activity, and sun exposure [39, 40]. Our study includes community-based people of European descent, living in a similar latitude (all living in Melbourne); we have performed sex-specific analysis, adjusted for age, BMI, physical activity; and measurements of 25(OH)D were corrected for season of blood collection. Thus any misclassification associated with single time measurement of 25(OH)D and total hip replacement for OA is likely to be non-differential in nature. We could not control for occupational activity, since the MCCS did not have detailed information such as heavy lifting and climbing which are established risk factors for hip OA. We defined hip OA based on joint replacement which reflects end-stage joint disease, and thus less severe OA was not captured. It is possible that there is residual or unmeasured confounding, for example activities that might influence hip injury. We did not have accurate hip replacement data prior to 2001. It is possible that hip replacement occurring before 2001 might represent more rapidly progressive disease, yet we were only able to assess hip replacement for OA occurring after this time and cases in the first few years of follow-up were missed, this would have resulted in non-differential misclassification. Although it has been suggested that people who uses painkillers i.e. paracetamol, non-steroidal anti-inflammatory drugs (NSAIDs) and acetylsalicylic acid might have lower levels of vitamin D [41], the Danish Osteoporosis Prevention Study could not identify any difference in circulating levels of 25(OH)D between participants who were using pain killers and those who were not using pain killers (24.3 (11.4) ng/ml vs 25.2 (12.3) ng/ml; *p* = 0.34) [42]. Furthermore, if pain killers were an explanatory factor in this study, we would have found more THR in those who had lower levels of circulating 25(OH)D which was not the case. Data on vitamin D supplementation were lacking. However, at the time of MCCS recruitment (1990-1994), supplement use in Australia was uncommon (16% of MCCS participants reported using multivitamins at baseline), and this is unlikely to fully explain the observed association. Screening for vitamin D deficiency and vitamin D supplement use have increased since the early 2000s [43]. However, fluctuations in 25(OH)D measurements with differences between participants along time cannot be ruled out. Strengths of our study include its prospective cohort study design, validated and reliable measurement of circulating 25(OH)D concentrations [19], adjustment for important confounders, and the validation and completeness of AOA NJRR data on hip replacement for OA.

Internationally there is a major focus on targeting vitamin D deficiency in order to reduce the burden of osteoporotic fractures. However, the ideal concentration of 25(OH)D is still debated since some experts suggest concentrations less than 50 nmol/L are “insufficient” while others consider less than 75 nmol/L as “insufficient” [44–46]. There is increasing evidence that associations between 25(OH)D and chronic diseases, if they do exist, are non-linear. For example, in a meta-analysis, risk of cardiovascular disease decreased with increasing 25(OH)D concentration up to 60 nmol/L, above which there was no further decrease or increase [47]. In a randomized controlled trial of community-dwelling women aged 70 years or older, annual oral administration of high-dose cholecalciferol (vitamin D_3_) resulted in an increased risk of falls and fractures [48]. Taken together, these data highlight the need for further research to determine the optimal 25(OH)D concentrations for preventing fractures in different populations and reducing the risk of other chronic diseases.

## Conclusions

This study confirms our previous results that greater circulating 25(OH)D concentrations were associated with an increased risk of total hip replacement for OA in men but not in women. Further work is needed to understand the mechanisms underlying these findings. Our findings also highlight the need to determine the optimal levels of circulating 25(OH)D in order to reduce the risk of chronic diseases such as hip OA.

## Data Availability

The datasets used and/or analysed during the current study are available from the corresponding author on reasonable request.

## Abbreviations

25(OH)D: 25-hydroxyvitamin D
OR: Odds ratios
CI: Confidence intervals
OA: Osteoarthritis
MCCS: Melbourne Collaborative Cohort Study
AOA NJRR: Australian Orthopaedic Association National Joint Replacement Registry
SEIFA: Socio-Economic Indexes For Areas
BMI: Body mass index
LC-MS/MS: Liquid chromatography-tandem mass spectrometry
NIST: National Institute of Standards and Technology
DEQAS: Vitamin D External Quality Assessment Scheme
HR: Hazard ratio
